# DTI Image Registration under Probabilistic Fiber Bundles Tractography Learning

**DOI:** 10.1155/2016/4674658

**Published:** 2016-09-27

**Authors:** Zhe Guo, Yi Wang, Tao Lei, Yangyu Fan, Xiuwei Zhang

**Affiliations:** ^1^School of Electronics and Information, Northwestern Polytechnical University, Xi'an 710072, China; ^2^School of Computer Science, Northwestern Polytechnical University, Xi'an 710072, China; ^3^College of Electrical & Information Engineering, Shaanxi University of Science & Technology, Xi'an 710021, China

## Abstract

Diffusion Tensor Imaging (DTI) image registration is an essential step for diffusion tensor image analysis. Most of the fiber bundle based registration algorithms use deterministic fiber tracking technique to get the white matter fiber bundles, which will be affected by the noise and volume. In order to overcome the above problem, we proposed a Diffusion Tensor Imaging image registration method under probabilistic fiber bundles tractography learning. Probabilistic tractography technique can more reasonably trace to the structure of the nerve fibers. The residual error estimation step in active sample selection learning is improved by modifying the residual error model using finite sample set. The calculated deformation field is then registered on the DTI images. The results of our proposed registration method are compared with 6 state-of-the-art DTI image registration methods under visualization and 3 quantitative evaluation standards. The experimental results show that our proposed method has a good comprehensive performance.

## 1. Introduction

Diffusion Tensor Imaging (DTI) is a Magnetic Resonance Imaging (MRI) technique which measures diffusion properties of water molecules in tissue to gained neural bundle images, which cannot be obtained by other imaging modalities [1]. It captures vital information that import for vivo investigation of white matter and connectivity alterations, thus playing an increasingly significant role* in vivo* studies of anatomical structure and functional connectivity in the brain regions [2]. DTI image registration is an essential step for diffusion tensor image analysis. DTI registration is involved in many clinical diagnoses of disease diffusion tensor image analysis; all need image registration techniques [3]. For ordinary medical image registration, the corresponding points of two images will be transformed to have the exact consistency on the space position and the anatomical structure by space transformation; the registration process is essentially a multiparameter optimization problem [4]. Tensor image registration will encounter many ordinary medical image registration problems but also includes some special difficulties due to the particularity of the DTI data.

According to the object of the registration algorithm, the existing DTI image registration algorithms can be divided into three categories: the scalar image based registration algorithm, the tensor image based registration algorithm, and the fiber bundle based registration algorithm [5]. The scalar image based registration algorithm has low computational complexity; however, as this algorithm does not make full use of all the directions and structure information of the DTI images, it will lose some important data in the registration process. The tensor image based registration algorithm should ensure the consistency of the tensor direction and anatomical structure before and after the transformation. Fiber bundle based registration algorithm directly uses white matter fiber bundles for registration and can avoid the estimation error of DTI direction and hence improve accuracy and robustness of registration. Therefore, in recent years, this kind of registration algorithm becomes the mainstream of diffusion tensor magnetic resonance image registration method.

In the fiber bundle based registration method, fiber tracking is a very important step, and the correctness of fiber tracking directly affects the accuracy of the registration [6]. The neural fiber tracking technique based on DTI can be roughly divided into two categories: deterministic fiber tracking technique and probabilistic fiber tracking technique. As the diffusion tensor of the voxel is sensitive to noise, the result of the deterministic fiber tracking will be affected by the noise also. Furthermore, due to the effect of volume, there is more than one fiber beam in the unit voxel. For the area including two or multiple nerves and fibers cross through, the accuracy of deterministic fiber tracking is not high [7]. Because of the introduction of probability statistics method, the probabilistic fiber bundle tracking technique can solve the problem of volume effect and noise interference [8].

Probabilistic tractography uses a deterministic streamline algorithm to generate thousands of trajectories by Monte Carlo methods. The directions of the line segments are repeatedly sampled from a Bayesian posterior distribution [8]. The probability of a trajectory to the selected sample voxel is then defined as the number of virtual fibers passing a voxel. Probability distribution based on* a priori* assumptions about the form of the uncertainty in the data is used in most probabilistic tractography methods. Nevertheless, since a parametric description of subject artifacts is generally unavailable, the uncertainty is modeled without considering the artifacts interference.

To resolve this disadvantage, a typical active sample selection learning method called bootstrap [9] has been incorporated in probabilistic tractography [10]. Bootstrap method is nonparametric procedure which assesses the measurement uncertainty of parameters without the assumption of a noise model and the acquirement of large amount of datasets [11]. Consequently, the local directions are derived by resampling from the acquired data itself instead of a probability distribution.

Based on the above analysis, in this paper, we proposed a DTI image registration method under probabilistic fiber bundles tractography learning. We improve the residual error estimation step in bootstrap method used in active sample selection learning for the probabilistic tractography. Our method assumes that, in the case of independent and identically distributed error, the residuals can be adjusted and the error model is then modified by using finite sample set, therefore, improving the study ability of samples. Subsequently, the tracked fiber bundles can be registered by using symmetric image standardization registration algorithm. The results of our proposed registration method are compared with 6 state-of-the-art DTI image registration methods under visualization and 3 quantitative evaluation standards [12] for the comprehensive analysis. The experimental results show that our proposed registration method under probabilistic fiber bundles tractography learning has a good comprehensive performance.

## 2. Related Works

DTI registration methods are mainly divided into three parts by the processing object: the scalar image based registration algorithm, the tensor image based registration algorithm, and the fiber bundle based registration algorithm.

### 2.1. Scalar Image Based Registration

Scalar image based registration methods convert tensor images into scalar images, for example, fractional anisotropy (FA) images, by rotationally invariant measures, and then perform registration on the scalar images. Studholme et al. [13] proposed the rigid registration method (denoted as Rigid), which was based on normalized mutual information. This method was commonly used in therapy planning, clinical diagnosis, and automatic clinical image registration as a rough registration. Multiresolution elastic matching algorithm (denoted as Elastic) [14] and multiresolution B-spline method [15] were proposed successively and applied to the registration of diffusion tensor images; the latter was proved to have the high geometric fidelity [16]. Consequently, Andersson et al. [17] developed B-spline registration based on sum-of-squared differences (denoted as FSL), the regularization that was based on membrane energy, and a multiscale Leven berg-Marquardt minimization avoided the local minimum value. Soon after, affine image coregistration technique (denoted as Affine) [18] was performed in some cases to align images before the application of higher order registration. In 2008, the literature [19] discussed normalized mutual information criterion, in which the symmetrized Kullback-Leibler divergence was used to improve fluid registration of diffusion tensor images. This algorithm was diffeomorphic and reversible consistency but performed badly on smoothness and was time-consuming. Recently, Hufnagel et al. [20] mentioned the block-matching algorithm in his article. In this method, the determined sparse displacement vector field was used for nonlinear transformation parameters estimation.

Diffeomorphic mapping is a smooth spatial transform, in which the topology of the images is preserved, as well as voxel correspondence based on the second-order tensor field of Riemannian manifold. It was combined with Lie group [21, 22] structure to perform relatively simple calculation. Without the Riemannian manifold measure, directly computing differences between tensors with Euclidean space would lead to the “tensor swelling effect” [23] and could not guarantee reversible consistency of the transformation. Diffeomorphic mapping can avoid the “tensor swelling effect,” guarantee the reversible consistency of the transformation and the smoothness, and enhance the computational efficiency and registration precision.

Based on the above advantages, Cao et al. [24] developed a large diffeomorphic registration algorithm for vector fields. Due to computational difficulties, this algorithm was not applied widely. However, this method was a successful foundation for differential homeomorphism registration method. In 2008, Vercauteren et al. [25] proposed the symmetric log-domain diffeomorphic registration method. The parameterization of diffeomorphic transformations was done completely in log-domain, based on stationary velocity field and Lie group structure, which guaranteed the invertibility of deformation and had access to the true inverse transformation. Almost simultaneously, Avants et al. [26] developed a symmetric image normalization method (denoted as SyN). The Euler-Lagrange equation was used for the optimization. In 2009, Vercauteren et al. [27] proposed an efficient nonparametric diffeomorphic image registration algorithm. It optimized the entire space of displacement fields based on Thirion's demons algorithm [28]. In 2010, the literature [29] compared symmetric log-domain diffeomorphic registration and asymmetric log-domain diffeomorphic registration. The results showed that the former has good reversible consistency by catching transformation information.

### 2.2. Tensor Image Based Registration

The registration of tensor image is more difficult than scalar image based registration. One reason is multidimensionality of the data. Another is that anatomical structure has changed after image transformations. We need to ensure the tensor orientations to keep consistence with the anatomy.

In 2003, Park et al. [30] proposed multichannel DTI registration method based on the Demons algorithm. The whole diffusion tensor and various features of tensor were used in this algorithm and this improved the quality of registration. But the tensor reorientation was not explicitly optimized and only applied tensor reorientation iteratively. In 2006, Zhang et al. [3] proposed diffeomorphic deformable tensor registration (named as DTI-TK). This method measured tensor similarity as a whole and enabled explicit optimization of tensor reorientation without additional correction to tensor orientation. In 2009, Yeo et al. [31] proposed exact finite-strain diffeomorphic registration, which combined exactly finite strain reorientation with the object function of Demons. This algorithm was reversible consistency and achieved significantly better registration with the exact gradient. However, the tensor reorientation was not optimized explicitly. In 2009, Yap et al. [32] proposed tensor image morphing for elastic registration. This algorithm leveraged tensor regional distributions and local boundaries directly and was improved by utilizing automatic detecting structure characteristics and thin-plate-spline (TPS) [33]. Recently, DTI-TK was improved in different ways, such as utilizing various tensor characteristics and orientation features with neighborhood interpolation [34], combining tract and tensor features [35], and also extending Statistical Parametric Mapping to reduce the computation complexity [36].

### 2.3. Fiber Bundle Based Registration

With direct registration of fiber, we can avoid the estimation error of DTI direction and improve the accuracy as well as robustness of the registration. In 2007, Mayer and Greenspan [37] proposed direct registration based on white matter (WM) fiber where fibers were represented as 3D points to be registered. This algorithm adopted an iterative closest fiber approach, and each iteration estimated the 12-parameter affine transformation. However this method was time-consuming. In 2010, Shadmi et al. [38] presented piecewise affine registration of fiber. The registration of fiber was considered as a problem of probability density estimation. The energy function was optimized by the gradient descent method and evaluated by residual mean square error. The algorithm made full use of fiber orientation, so it improved accuracy and robustness of the registration. In 2010, Zvitia et al. [39] proposed registration of WM fibers by Adaptive-Mean-Shift (AMS) and Gaussian Mixture Modeling (GMM). The fibers were projected into a high dimensional feature space based on 3D coordinates. The fiber modes were produced by the AMS, and the GMM of fibers was obtained by Gaussian distribution. The registration of WM fibers depended on the alignment of two GMMs.

Compared to the deterministic fiber tracking technology, probabilistic tractography technique can more reasonably trace to the structure of the nerve fibers and in a certain extent overcome the internal defect of the single tensor model. Since the probability statistics method is introduced, probabilistic tractography can effectively reduce uncertainty of tracking results by noise and other environmental factors and thus has better performance of antinoise interference. But there are few researches on the DTI image registration based on the probabilistic fiber bundles tractography.

To improve the efficiency of DTI image registration, we proposed a DTI image registration method under probabilistic fiber bundles tractography learning. We first get the distribution of the whole brain white matter fiber bundles based on probabilistic tractography. Then, the tracked fiber bundles can be registered by using symmetric image standardization registration algorithm, and the calculated deformation field acts on the DTI images, finally implementing the accurate DTI images registration. For the experiments, we compared our method with the state of the art methods under visualization and three quantitative evaluation standards and gave a comprehensive analysis.

Our method is innovative in the following two aspects:

1. Using fiber bundles tracked by probabilistic tractography to calculate the deformation field of DTI image registration: Registration based on white matter fiber bundles can avoid the estimation error of DTI direction. Furthermore, probabilistic tractography technique can more reasonably trace to the structure of the nerve fibers and can effectively reduce uncertainty of tracking results by noise and other environmental factors.
2. Improving the residual error estimation step in bootstrap method used in active sample selection learning for the probabilistic tractography: Our method assumes that, in the case of independent and identically distributed error, the residuals can be adjusted and the error model is then modified by using finite sample set, therefore, improving the study ability of samples.

## 3. Probabilistic Fiber Bundles Tractography Learning

### 3.1. Probabilistic Tractography

Given a brain diffusion MRI image, the DTI can be modeled as a simple diffusion with a Gaussian profile [40]:

(1) $$\begin{array}{c} G\left(\;x;D,t\;\right)={\left(\;{\left(\;4\pi t\;\right)}^{3}det\left(D\right)\;\right)}^{-1/2}exp\left(\;\frac{-x^{T}D^{-1}x}{4t}\;\right), \end{array}$$

where *D* is the diffusion tensor, *t* is the diffusion time, and *x* describes the element of the MRI image.

To sample the ellipsoid structure based on probability distribution, the 3 × 3 diffusion tensor *D* needs to be solved. The estimation of the diffusion coefficients of the tensor *D* can be implemented by six independent measurements along noncollinear gradient orientations. The solution to *D*, using singular value decomposition (SVD), identifies a new basis system describing the diffusion profile at each voxel using eigen values *λ*_1_,  *λ*_2_, and *λ*_3_ and the corresponding eigen vector **e**_1_,  **e**_2_, and **e**_3_ that indicate the preferred direction of water diffusion.

Probabilistic tracking algorithm devised by Friman et al. [8] is based on a Bayesian inference and estimation scheme. Due to noise or complex fiber architectures, uncertainties of probability are not disregarded but captured in the model itself, in form of the posterior distribution at each voxel. Given a source region *A* and a target region *B*, the probability of connectivity between *A* and *B* is given as

(2) $$\begin{array}{c} p\left(\;A⟶B\;|\;D\;\right)=\overset{\infty }{\underset{n=1}{\sum }}\overset{}{\underset{\Omega_{AB}^{n}}{\int }}p\left(\;n\;\right)p\left(\;\upsilon_{1:n}\;|\;D\;\right), \end{array}$$

where *p*(*υ*_1:*n*_∣*D*) is the probability of the fiber path going from *A* to *B*, given the diffusion tensor *D*, and *υ* represents a voxel. *Ω*_*AB*_^*n*^ represents the sampling space of the connectivity between *A* and *B* of path length 1 through *n*.

In order to make (2) analytically solvable, a rejection sampling strategy can be employed. Specifically, a large number of sampled fiber paths starting from region *A* are drawn randomly, and the probability of the path between *A* and *B* is then evaluated. These random paths need to be found by working at each step of the path up until the predetermined length *n*. We assume these steps are unit length vectors and only depended on previous step direction. Under this assumption, the posterior distribution at each step is calculated based on the diffusion data *D*. This distribution can be described in terms of Bayes theorem as

(3) $$\begin{array}{c} p\left(\;{\hat{\upsilon }}_{i},\theta \;|\;{\hat{\upsilon }}_{i-1},D\;\right)=\frac{p\left(D\;|\;{\hat{\upsilon }}_{i},\theta \right)p\left({\hat{\upsilon }}_{i}\;|\;{\hat{\upsilon }}_{i-1}\right)p\left(\theta \right)}{p\left(D\right)}, \end{array}$$

where $p\left(D|{\hat{\upsilon }}_{i},\theta \right)$ represents the likelihood distribution using a constrained model based on a Gaussian diffusion profile at the current point. $p\left({\hat{\upsilon }}_{i}|{\hat{\upsilon }}_{i-1}\right)$ is the prior to indicate that the current point depends on the direction of the previous step. The nuisance priors, *p*(*θ*), are the parameters of the Gaussian profile modeled as dirac priors which can significantly save computation time. *p*(*D*) is the normalizing constant. Those expressions combined together give the probability distribution at ${\hat{\upsilon }}_{i}$ over a unit sphere.

### 3.2. Active Sample Selection Learning

In the probabilistic tractography, sample selection is a very important step. Through the sample selection step, a large number of samples describing the fiber paths starting from region *A* can be obtained, and the probability density function of the path between *A* and *B* is then estimated according to a nonparametric procedure. After that, the probabilistic tractography can be completed based on a Bayesian inference and estimation scheme.

Sample selection learning is one kind of learning methods which learn from the environment to obtain a number of concept related examples and derive general concept after the induction. Bootstrap [9] is a typical active sample selection learning method which includes a nonparametric procedure to estimate the probability density function (PDF), by randomly selecting individual measurements, with replacement, from a set of repeated measurements, thus generating many bootstrap samples [10].

Having observed a random sample *V* = (*υ*_1_,…, *υ*_*n*_) with size *n*, from a distribution with cumulative density function *F*,

(4) $$\begin{array}{c} F⟶\left(\;\upsilon_{1},\ldots ,\upsilon_{n}\;\right), \end{array}$$

the empirical distribution function $\hat{F}$ is then defined to be the discrete distribution that puts probability 1/*n* on each *υ*_*i*_,  *i* = 1,…, *n*. The arrow notation (→) indicates that the sample values are outcomes of random variables with independent and identically distributed attribute, each with distribution function *F*, that is, *υ*_*i*_ $\underset{\tilde{\;\;}}{i.i.d}$ *F* [41]. A bootstrap sample is a random sample of size *n* drawn from $\hat{F}$, denoted as *V* ^*∗*^ = (*υ*_1_^*∗*^, *υ*_2_^*∗*^,…, *υ*_*n*_^*∗*^), obtained by

(5) $$\begin{array}{c} \hat{F}⟶\left(\;\upsilon_{1}^{*},\upsilon_{2}^{*},\ldots ,\upsilon_{n}^{*}\;\right). \end{array}$$

The star notation in the upper right corner of *V* ^*∗*^ indicates that *V* ^*∗*^ is not the actual data set *V*, but a randomized version of *V*. These significant amounts of bootstrap samples enable us to estimate the sampling distribution statistics for making inferences about a population parameter *φ*. If estimate is denoted as $\hat{\varphi }=s\left(V\right)$, for each bootstrap sample, a bootstrap replication of $\hat{\varphi }$ can be computed by [41]

(6) $$\begin{array}{c} {\hat{\varphi }}^{*}=s\left(\;V^{*}\;\right). \end{array}$$

A collection of bootstrap replication will provide us with the information needed to obtain the sampling distribution estimation of $\hat{\varphi }$.

The wild bootstrap (WB) proposed originally by Wu [42] is suited when the residuals of regression model exhibit heteroskedasticity. The observations in this case, *V* = [(*x*_1_, *y*_1_),…, (*x*_*n*_, *y*_*n*_)], are assumed to be instances of bivariate random variable (**X**, **Y**). **X** is a *R* ^*d*_**X**_^-valued predictor random variable and **Y** is a *R* ^*d*_**Y**_^-valued response random variable. If $\hat{\ell }\left(x\right)$ is an estimate of the regression function *ℓ*(*x*) = *E*(**Y**∣**X** = *x*) of **Y** on **X**, WB resamples the residuals by assuming the “true” residual distribution is symmetric. For the least square regression, the finite sample is used to replace the residuals $r_{i}=y_{i}-\hat{\ell }(x_{i})$ by the factor (1 − *h*_*i*_)^−1/2^,  *i* = 1,…, *n*, where *h*_*i*_ is the *i*th diagonal element from the hat matrix of the ordinary least squares solution.

Our method assumes that, in the case of independent and identically distributed error, the residuals can be adjusted and the error model is then modified by using finite sample set. Consequently, our method generates each bootstrap sample using

(7) $$\begin{array}{c} V^{*}=\left[\;\left(\;x_{1},\hat{l}\left(\;x_{1}\;\right)+{\tilde{r}}_{1}^{*}\;\right),\ldots ,\left(\;x_{n},\hat{l}\left(\;x_{n}\;\right)+{\tilde{r}}_{n}^{*}\;\right)\;\right], \end{array}$$

where ${\tilde{r}}_{i}^{*}={\tilde{r}}_{i}+\gamma^{-1}h_{i}\psi ({\tilde{r}}_{i})$,  *γ* = ∫*ψ*(*x*)*f*(*x*)*dx*,  *f* is the density function of *r*_*i*_,  *ψ* is the score function, and *h*_*i*_ = *x*_*i*_^*T*^(∑_*k*_ *x*_*k*_ *x*_*k*_^*T*^)^−1^ *x*_*i*_,  *k* ≪ *n*. The tildes denote the corrected residuals.

## 4. Materials and Registration Method

### 4.1. Ethical Standards

*Ethical Approval*. All procedures performed in studies involving human participants were in accordance with the ethical standards of the institutional and/or national research committee and with the 1964 Helsinki declaration and its later amendments or comparable ethical standards.

*Informed Consent*. Informed consent was obtained from all individual participants included in the study.

### 4.2. Materials

*Diffusion MRI Data*. The open accessed IXI dataset from Hammersmith Hospital of London was used (http://www.brain-development.org/). A 3 Tesla Philips MRI scanner was used to scan the healthy subjects. With spatial resolution 1.7409 × 1.7355 × 1.9806 mm, the volume data of head is 128 × 128 × 64 voxels. Diffusion weighted images are along unique gradient directions with* b* = 1000 s/mm^2^ (repetition time = 11894.44 ms; echo time = 51 ms). More parameter information can be found at the website.

**Figure 1:**
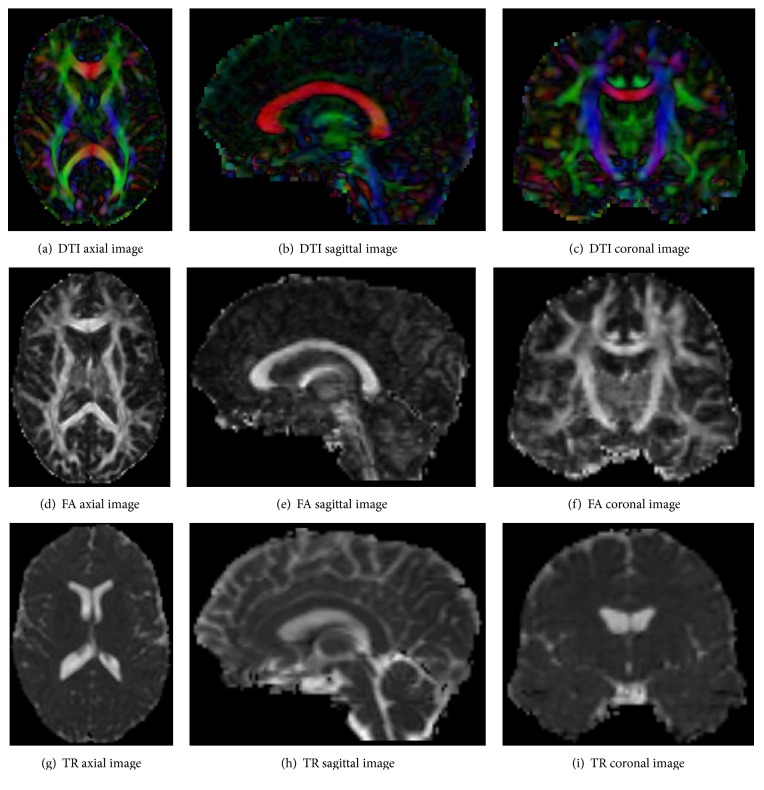
2D views of the template (the color images are encoded as follows: red for left-right, green for anterior-posterior, and blue for inferior-superior).

*Template and Subject*. In this paper, 10 data were chosen randomly as subjects (5 male, average age = 51.586 years, min age = 30.89 years, and max age = 63.68 years; 5 female, average age = 51.512 years, min age = 33.76 years, and max = 74.01 years) and also another data was chosen as the template (male, age = 37.83 years). The template is shown in Figure 1. The white matter area in FA is obviously highlighted in Figures 1(d), 1(e), and 1(f). TR means the trace of diffusion tensor.

### 4.3. Preprocessing

Brain Extraction Tool (BET) in FMRIB software Library was used to extract brain tissue for each subject and template. The mask used for skull stripping was generated from each subject or template individually and checked manually. Before tensor estimation, diffusion weighted images (DWIs) in 15 diffusion gradient directions were eddy-current corrected with FMRIB software Library.

### 4.4. Registration Method

In this paper, the symmetric image standardization algorithm (also called symmetric image normalization, SyN) proposed by Avants et al. [26] is used to register the tracked fiber bundles. In this method, the cross-correlation is made as similarity criterion, and the Euler-Lagrange equation is used for algorithm optimization. In this way, the diffeomorphism transformation can be decomposed into two parts and also ensures the reversible consistency of the spatial transform.

In the spatial domain of fiber bundles *Ω*, if the diffeomorphism transformation function is *φ*, affine transformation of fiber bundle can be noted as

(8) $$\begin{array}{c} \varphi \left(\;\partial \Omega \;\right)=A\left(\;Id\;\right), \end{array}$$

where **A**(**I** **d**) is the affine transformation, and the symmetrical and time varying velocity field is

(9) $$\begin{array}{c} \frac{d\varphi \left(x,t\right)}{dt}=v\left(\;\varphi \left(\;x,t\;\right),t\;\right). \end{array}$$

Through the integration of time and smooth velocity field, the diffeomorphism transformation *φ* can be obtained. *φ* can be decomposed into two parts: *φ*_1_ and *φ*_2_, transformed to the middle point along the geodesic line, for the fiber bundles to be registered and standard fiber bundle templates, respectively. The parameters are

(10) υx,t=υ1x,tt∈0,0.5,ux,t=u2x,1−tt∈0.5,1,

the corresponding fiber bundle set can be obtained by integral transform, and the similarity measurement is

(11) $$\begin{array}{c} {\left|\;\varphi_{1}\left(\;x,t\;\right)I-\varphi_{2}\left(\;x,1-t\;\right)J\;\right|}^{2}. \end{array}$$

The Euler-Lagrange equation is then used for algorithm optimization. For computation from the fiber bundle to be registered to the standard template fiber bundle or from the standard template fiber bundle to the fiber bundle to be registered, the path is the same (**I**⇔**J**), which ensures the reversible consistency. The formula of reversible consistency can be described as

(12) φ1−1φ1=Id,φ2−1φ2=Id.

SyN algorithm can deal with both small and large deformation. The results will not change by the input data order, and the diffeomorphic mapping ensures the precision of the reversible consistency transform.

### 4.5. Evaluation Criteria

#### 4.5.1. Dyadic Coherence *κ* [43]

After the eigen decomposition of the diffusion tensor, the eigenvalues could be denoted in descending order as *λ*_1_ > *λ*_2_ > *λ*_3_, and the corresponding eigenvectors are denoted as **e**_1_,  **e**_2_, and  **e**_3_. Dyadic coherence describes the variability in the dominant diffusion direction. For each voxel, the dyadic coherence is defined as

(13) $$\begin{array}{c} \kappa =1-\sqrt{\frac{\beta_{2}+\beta_{3}}{2\beta_{1}}}, \end{array}$$

where *κ* measures the variability of eigenvectors, which ranges from 0 to 1 (0 for randomly and 1 for identically oriented directions). *β*_*j*_  (*j* = 1, 2, 3) is the eigenvalue of the mean dyadic tensor [38]. The higher value for the dyadic tensor will represent better eigenvector alignment and higher fiber alignment accuracy.

#### 4.5.2. Overlap of Eigenvalue-Eigenvector (OVL) [44]

The overlap of eigenvalue-eigenvector pairs is defined as

(14) $$\begin{array}{c} OVL=\frac{1}{N}\overset{N}{\underset{i=1}{\sum }}\frac{\sum_{j=1}^{3}\left(\lambda_{j}^{i}\lambda_{j}^{\prime i}{\left(e_{j}^{i}\cdot e_{j}^{\prime i}\right)}^{2}\right)}{\sum_{j=1}^{3}\lambda_{j}^{i}\lambda_{j}^{\prime i}}, \end{array}$$

where *λ*_*j*_^*i*^,  **e**_*j*_^*i*^,  *λ*_*j*_^′*i*^, and  **e**_*j*_^′*i*^ are the *j*th eigenvalue-eigenvector pairs from the *i*th subject and the template tensors, respectively. The value of OVL is more closer to 1; the alignment of tensor orientation and fibers is better.

#### 4.5.3. Cross-Correlations (CC_*x*_) [45]

The cross-correlations of the WM voxels between subjects and template are computed by using the FA and TR:

(15) $$\begin{array}{c} CC_{x}=\frac{\sum_{v}X_{1}\left(v\right)X_{2}\left(v\right)}{\sqrt{\sum_{v}X_{1}\left(v\right)X_{1}\left(v\right)\sum_{v}X_{2}\left(v\right)X_{2}\left(v\right)}}, \end{array}$$

where *v* indexes over all the voxels. *X*_1_(*v*) and *X*_2_(*v*) are scalar images derived from DTI and could be replaced by FA or TR. The value ranges between 0 and 1. The higher cross-correlation describes the higher similarity between two maps.

## 5. Experimental Results

In order to test the performance of the proposed registration method based on probabilistic fiber bundles tractography learning, in this paper, we compare our method with 6 state-of-the-art methods, which are 5 scalar based methods: Rigid [13], Affine [18], Elastic [14], SyN [26], FSL [17], and one tensor based method: DTI-TK [3]. Dyadic coherence *κ*, overlap of eigenvalue-eigenvector (OVL), and cross-correlations (CC_*x*_) are used as three evaluation criteria. Maps and empirical cumulative distribution functions (CDFs) are used for illustrating 7 registration algorithms. CDF is probability of variable less than or equal to a certain number; that is, *F*(*x*) = *p*(*X* ≤ *x*), where *P* is probability.

### 5.1. Fiber Bundles Tracking Results

**Figure 2:**
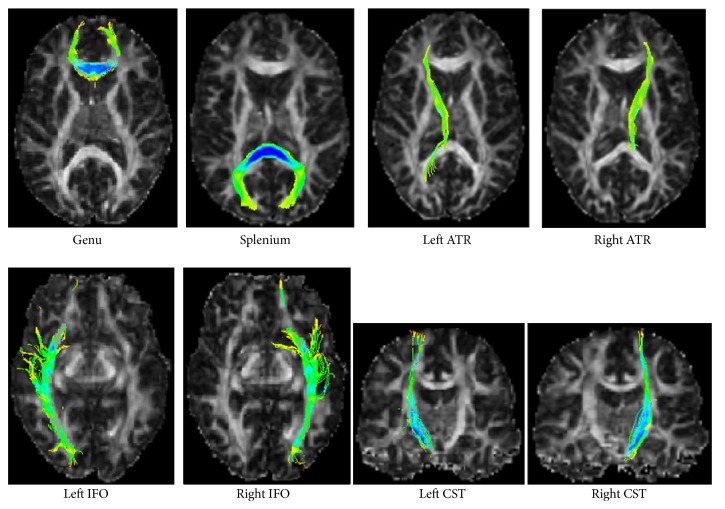
Fiber bundles tracking results (8 ROIs is Genu of the Corpus Callosum (Genu), Splenium of the Corpus Callosum (Splenium), Left Anterior Thalamic Radiations (ATR), Right ATR, Left Inferior Frontooccipital Fasciculi (IFO), Right IFO, Left Corticospinal/Corticobulbar Tracts (CST), and Right CST).

**Figure 3:**
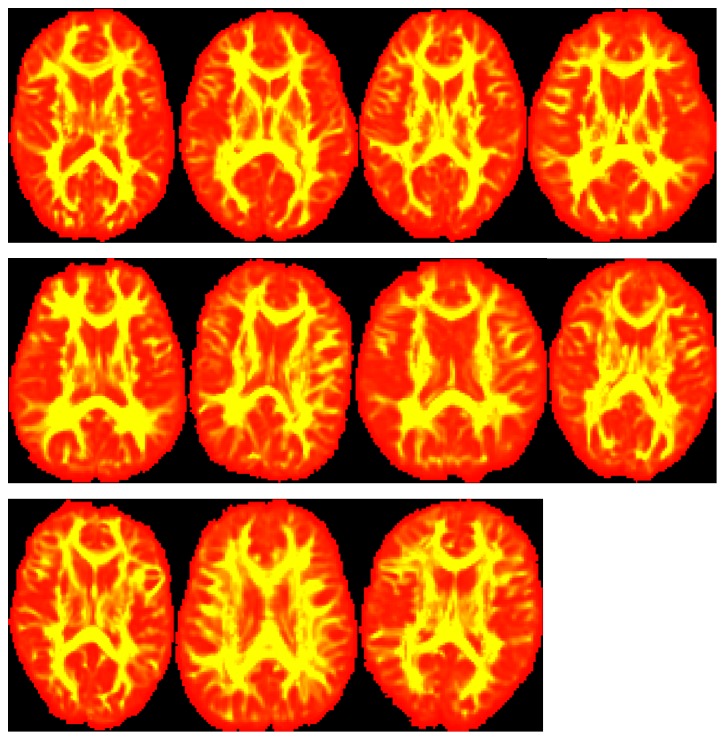
Global display of fiber bundles tracking results.

The result of fiber bundles tracking will affect the accuracy of the following registration method as tracking result is the input of registration step; as a result, fiber bundles tracking is an important step in the whole algorithm system.  Figure 2 gives fiber bundles tracking results of 8 regions of interest (ROIs) by the probabilistic fiber tracking algorithm proposed in this paper.  Figure 3 shows the global display of fiber bundles tracking results for 11 experimental data. From Figures 2 and 3, it can be seen that our proposed probabilistic fiber bundles tracking method has the ability to tracking white matter fiber bundles of diffusion MRI image accurately.

### 5.2. Comparison of Registration Results

**Figure 4:**
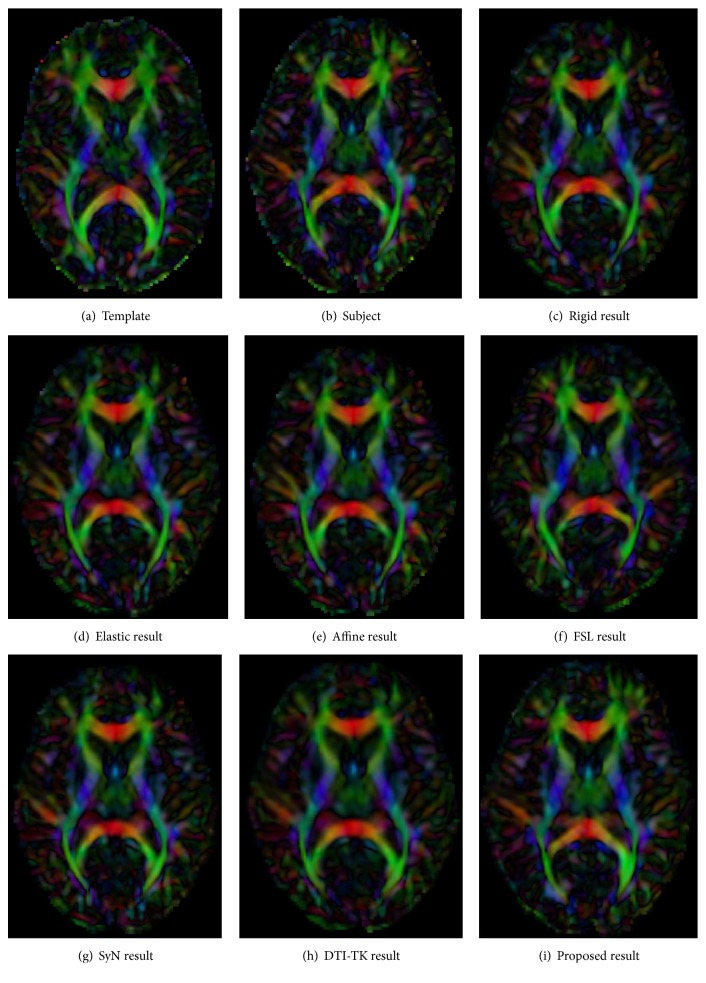
The results of 7 registration algorithms.

In this section, we test the registration effectiveness by visualization. The results of 7 registration algorithms are shown in Figure 4. In Figure 4, images of all the registered datasets were visualized, which could give qualitative results. From the visual results, our proposed method keeps the distribution character of subject white matter fiber bundles and also gets the better matching results with the template.

### 5.3. Comparison by Dyadic Coherence *κ*

**Figure 5:**
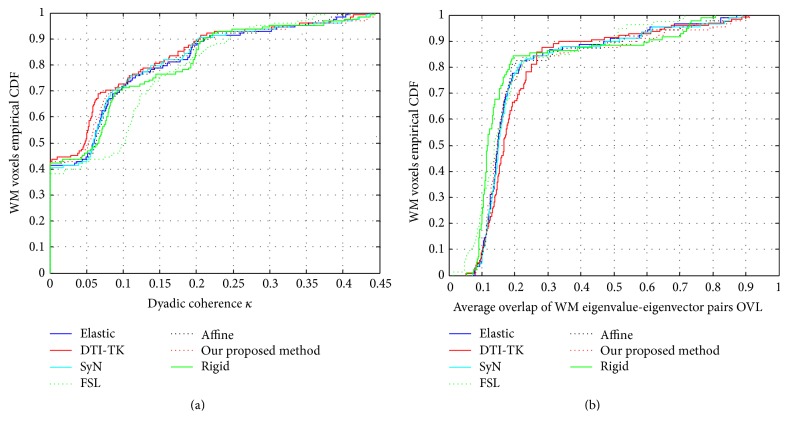
The empirical CDFs of dyadic coherence and OVL. (a) is the empirical CDFs of dyadic coherence; (b) is the empirical CDFs of OVL.

The higher dyadic coherence value indicates better eigenvectors alignment and anatomical structure consistency. The empirical CDFs of dyadic coherence are presented in Figure 5(a). From the empirical CDFs of dyadic coherence, *κ* of DTI-TK is the biggest, which indicates the highest anatomical structure consistency. Our proposed method gets the second ranking, only slightly worse than the DTI-TK method and much better than the existing Rigid, Affine, Elastic, SyN, and FSL methods, while, with the increase of *κ* value, the empirical CDFs of FSL increase rapidly and even exceed the DTI-TK and ours, but the overall empirical CDFs shock more seriously, which means the algorithm performance is not stable.

### 5.4. Comparison by Overlap of Eigenvalue-Eigenvector (OVL)

A higher OVL values represents a greater correspondence in anatomical structure between subjects. The empirical CDFs of OVL are presented in Figure 5(b). From Figure 5(b), it can be seen that when the OVL value is low, Rigid, FSL, and our proposed method show the better performance. As OVL increases, DTI-TK, Elastic, FSL, and our proposed method got the better performance. Considering the changing curve of OVL synthetically, DTI-TK, Elastic, and our proposed method are three stable methods. Our method is better than DTI-TK and quite equal to Elastic.

### 5.5. Comparison by Cross-Correlation of Diffusion (CC_*x*_)

**Figure 6:**
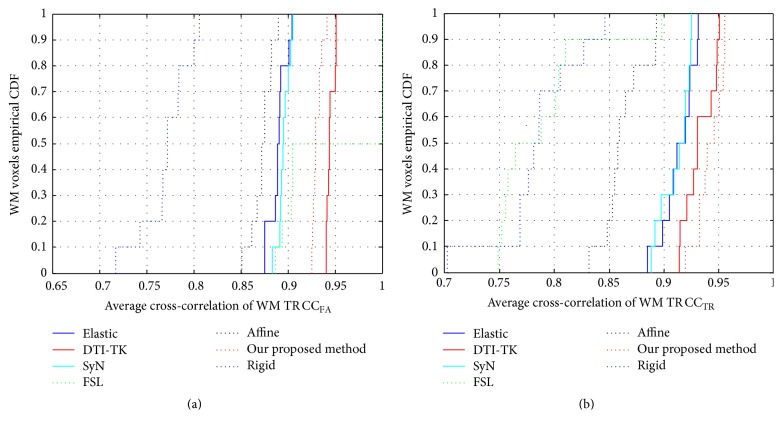
The empirical CDFs of cross-correlation. (a) is the empirical CDFs of the cross-correlations for FA; (b) is the empirical CDFs of the cross-correlations for TR.

For the cross-correlation, higher value represents the higher similarity between two maps. The empirical CDFs of cross-correlation for FA and TR are presented in Figures 6(a) and 6(b), respectively. From the empirical CDFs of CC_FA_, DTI-TK and our method show the top two highest performance, our method only slightly worse than the DTI-TK method. From the empirical CDFs of CC_TR_, our method is the best, which indicates highest image similarity.

From three evaluation criteria and visualization experimental results, our proposed method shows a high comprehensive performance. DTI-TK method is the currently recognized best registration method; our method shows the quite equal comprehensive performance. DTI-TK is a nonparametric, diffeomorphic deformable image registration, taking tensors as a whole and explicating the optimization of tensor reorientation. The disadvantage of the DTI-TK is that the image boundary is not smooth and the computing is complicated. Meanwhile, it only supports the affine transformation with the least parameters. Our method is based on the completely different algorithm theory; we completes the DTI Image registration method under probabilistic fiber bundles tractography learning. The distribution of the whole brain white matter fiber bundles is first obtained based on probabilistic tractography. Then, the tracked fiber bundles are registered by using symmetric image standardization registration algorithm, and the calculated deformation field acts on the DTI images. Those steps all have the advantages to improve the registration accuracy and robustness.

For the empirical CDFs of CC_TR_ and OVL, our method is better than DTI-TK. The experimental results show that the proposed method has a very good comprehensive performance and can be used for DT-MRI Image registration.

## 6. Conclusions

In this paper, we proposed a DTI Image registration method under probabilistic fiber bundles tractography learning, as the probabilistic tractography technique can more reasonably trace to the structure of the nerve fibers and in a certain extent overcome the internal defect of the single tensor model. We improved the residual error estimation step in bootstrap method used in active sample selection learning for the probabilistic tractography. The results of our proposed method were compared with 5 scalar based methods (Rigid, Affine, Elastic, SyN, and FSL) and one tensor based method (DTI-TK). The visualization and 3 quantitative evaluation standards were used to give a comprehensive analysis. The experimental results show that our proposed probabilistic fiber bundles tracking method has the ability to track white matter fiber bundles of diffusion MRI image accurately. Our registration method gives a quite equal comprehensive performance with DTI-TK, much better than the others. Consequently, our method can be used for accurate and efficient DTI image registration.
